# The effect of moral identity on facial emotion processing in adolescents with hearing loss: an event-related potentials study

**DOI:** 10.3389/fnins.2025.1559627

**Published:** 2025-03-26

**Authors:** Mengyao Jian, Yousong Hu, Jinlin Yang, Jun Chen

**Affiliations:** ^1^Department of Emergency Medicine, Institute of Disaster Medicine and Institute of Emergency Medicine, West China Hospital, Sichuan University, Chengdu, China; ^2^Department of Psychology, Chengdu Normal University, Chengdu, China; ^3^Sichuan Basic Education Research Center, Chengdu, China; ^4^Department of Gastroenterology and Hepatology, West China Hospital, Sichuan University, Chengdu, China; ^5^School of Psychology, South China Normal University, Guangzhou, China; ^6^Key Laboratory of Brain, Cognition and Education Sciences (South China Normal University), Ministry of Education, Center for Studies of Psychological Application, South China Normal University, Guangzhou, China; ^7^Guangdong Key Laboratory of Mental Health and Cognitive Science, South China Normal University, Guangzhou, China

**Keywords:** adolescents with hearing loss, adolescents with typical hearing, facial emotion processing, moral effect, event-related potentials

## Abstract

**Objective:**

This study investigates how the moral characters of others influence the recognition of facial emotional expressions in adolescents with hearing loss (HL), and compares these effects with those in adolescents with typical hearing (TH).

**Methods:**

A moral priming paradigm was employed to explore the neural mechanisms underlying facial emotion perception (happy, neutral, and angry) in different moral contexts (high moral, low moral). Event-related potentials (ERP) were utilized to assess brain responses.

**Results:**

Adolescents with TH evaluated emotional valence independently of moral context. In contrast, adolescents with HL judged faces with low moral levels more negatively. ERP analyses showed facial expression processing in adolescents with TH was not influenced by moral information, whereas adolescents with HL exhibited moral effects during both the middle (N2) and late stages (LPP) of processing.

**Conclusion:**

These findings underscore distinct neurocognitive mechanisms of emotion recognition in adolescents with HL and highlight the significant influence of moral identity on their emotional judgments.

## Highlights

- *Question*: how does moral identity affect facial emotion recognition in adolescents with hearing loss compared to typical hearing adolescents?
- *Findings*: adolescents with hearing loss exhibited greater interference from moral identity cues during facial emotion recognition, particularly in the N2 and LPP ERP components, while adolescents with typical hearing were less affected.
- *Importance*: these findings highlight the unique cognitive challenges faced by adolescents with hearing loss in social contexts and underscore the importance of developing tailored interventions to enhance their emotional recognition abilities.
- *Next Steps*: future research should explore the efficacy of cognitive training programs designed to improve inhibitory control and emotional processing in adolescents with hearing loss.

## 1 Introduction

Hearing loss (HL) affects approximately 115,000 children under the age of seven in China, with 30,000 newborns diagnosed annually (Liang and Mason, 2013). These children encounter significant difficulties in facial emotion recognition, a fundamental aspect of emotion processing, due to their limited access to auditory cues, which in turn hampers social communication (Başkent et al., 2023; Dammeyer, 2009; Calderon and Greenberg, 2003; Huang et al., 2024). The integration of moral and emotional cues in social cognition is particularly challenging for this population, yet remains underexplored.

Moral identity, defined as the internalization of moral values into one’s self-concept, plays a crucial role in shaping social behavior and empathy (Aquino and Reed, 2002; Hardy and Carlo, 2011). It facilitates emotion recognition by enhancing sensitivity to morally salient cues (Firestone and Scholl, 2016). The *moral pop-out effect* suggests that morally charged stimuli attract attention more effectively (Van Berkum et al., 2009; Gantman and Van Bavel, 2014, 2015), which may be particularly relevant for HL adolescents who rely primarily on visual cues for social interactions. However, the extent to which moral identity modulates emotion recognition in this population remains unclear.

Neuroscientific research highlights key event-related potential (ERP) components involved in facial emotion processing. The three-stage model outlines distinct neural mechanisms: (1) early perceptual detection (N1, P1) for physical stimulus characteristics (Ahmadi et al., 2018), (2) specialized facial feature analysis (N170, N2) for structural and emotional differentiation (Sun et al., 2017; Lee and Tong, 2024), and (3) higher-order emotional evaluation (P300, LPP) (Luo et al., 2010). Given the importance of later ERP components in integrating emotional and moral information, this model provides a strong foundation for investigating the influence of moral identity on facial emotion recognition in HL adolescents.

In line with this framework, peak amplitudes were used for N170 and N2 due to their relevance in early perceptual processing (N170) and cognitive control functions (N2), particularly during facial and moral judgments (Dai et al., 2014; Caharel and Rossion, 2021). In contrast, the mean amplitude was chosen for LPP, as it reflects sustained attention and emotional regulation, which are critical for evaluating emotionally salient stimuli (Luo et al., 2010; Luyster et al., 2019; Chen et al., 2023). To ensure accuracy in component identification, grand average ERP waveforms were visually inspected to define the measurement windows for each component.

Facial identity—encompassing both physical attributes (e.g., facial structure) and moral characteristics (e.g., trustworthiness, virtue)—has been shown to shape social cognition. The hierarchical model suggests that emotion recognition follows a sequential process, beginning with low-level visual feature analysis and progressing toward higher-order moral and emotional interpretations (Bruce et al., 1987). While early theories posited independent processing of identity and emotion (Bruce and Young, 1986), later research has demonstrated their interaction, particularly in ambiguous social contexts (Kaufmann and Schweinberger, 2004; Ganel and Goshen-Gottstein, 2004). This supports the *structural-reference theory*, which posits that facial structures serve as reference points for interpreting emotional cues (Ganel and Goshen-Gottstein, 2004). However, moral identity’s role in modulating this process remains unexplored.

Previous research has predominantly focused on the physical attributes of facial identity, such as gender and individual recognition, whereas moral identity processing involves distinct cognitive mechanisms. Unlike physical traits, which rely primarily on configural facial feature analysis, moral identity processing depends on semantic memory and contextual integration (Wieser and Brosch, 2012; Firestone and Scholl, 2016). The *moral pop-out effect* underscores the prioritization of morally salient cues in perception and decision-making (Gantman and Van Bavel, 2014, 2015), raising the question of whether moral identity similarly influences facial emotion recognition in HL adolescents.

Although researches indicates that moral identity enhances empathy and prosocial behavior (Yoder and Decety, 2014; Krettenauer and Victor, 2017; Liu et al., 2024), its role in mitigating deficits in emotion recognition—especially in individuals with HL—remains largely unexamined. Given that HL adolescents compensate for reduced auditory input by relying more on visual and contextual information (Hill et al., 2019; Choi et al., 2024), investigating whether moral identity can enhance their facial emotion recognition provides valuable insights into adaptive mechanisms in social cognition.

This study examines the influence of moral identity on facial emotion recognition in adolescents with HL, a relationship that has received limited empirical attention. Specifically, it tests whether moral identity serves as a compensatory mechanism for the emotion processing deficits commonly observed in this population.

To achieve this, we compare facial emotion recognition abilities between adolescents with HL and their typically hearing (TH) peers, focusing on how moral identity influences this process. By integrating neural (ERP) and behavioral measures, we assess whether moral identity enhances recognition accuracy and efficiency across these groups.

At the neural level, the study focuses on middle-stage (N170) and late-stage (LPP) ERP components, which are crucial for emotional integration and interpretation. These components provide insight into the mechanisms by which moral identity modulates facial emotion processing.

Methodologically, we employ an emotional conflict task with moral identity priming to investigate how moral identity influences emotion recognition at both behavioral and neural levels. By bridging moral psychology and cognitive neuroscience, this research provides a novel perspective on the interaction between moral identity and social–emotional cognition in adolescents with HL. Findings from this study will contribute to a deeper understanding of social development in HL adolescents and inform interventions aimed at improving social–emotional skills through identity-based approaches.

## 2 Methods

### 2.1 Participant

Using a convenience sampling method, 38 participants were recruited from three special education schools (*n* = 15) and one ordinary middle school (*n* = 22) in Guangdong Province, China. *A priori* power analysis, conducted using G*Power software (version 3.1.9.4), determined that a minimum sample size of 20 participants was required for the planned three-factor mixed analysis of variance. This calculation assumed a medium effect size (*f* = 0.25, equivalent to η^2^ = 0.062 or Cohen’s d = 0.52), a statistical power of 0.8, and a significance level of *p* < 0.05.

The study protocol was approved by the Ethical Review Board of South China Normal University (SCNU-PSY-2021-268). All experimental materials were designed with careful consideration of participants’ age characteristics and underwent ethical review to ensure appropriateness. To minimize potential psychological discomfort, the research team pre-assessed the materials before the experiment. Written informed consent was obtained from parents or guardians, who were informed of their right to withdraw at any time. Participants could also withdraw if they experienced discomfort, with the experimenter present to provide support. Upon completing the experiment, participants privately evaluated their task experience in a separate room to ensure confidentiality, with feedback limited to procedural aspects. Each participant received a 50 RMB Hongqi Supermarket voucher as appreciation. For the HL group, a trained instructor pre-reviewed materials and remained available outside the testing room to provide assistance or facilitate communication as needed.

Demographic information for participants is summarized in Table 1. To ensure homogeneity within the HL group, only participants with congenital or early-onset deafness (diagnosed before age three) who had not used hearing aids by age three were included. To confirm participants’ cognitive abilities, the Chinese version of the Raven Standard Progressive Matrices Test (Zhang and Wang, 1989), a widely accepted non-verbal intelligence assessment, was used. This test is particularly suitable for individuals with HL, including those in special education settings (Cn et al., 2018). All participants scored within the normal range on the Raven Standard Progressive Matrices Test (standard score > 5), with no significant IQ differences observed between the HL and TH groups, *F* (1, 35) = 0.302, *p* = 0.586. Additionally, all participants had normal or corrected vision and reported no mental health issues. None reported experiencing stressful life events within the 2 weeks preceding the experiment.

**Table 1:** Basic information of the participants.

	Adolescents with HL	Adolescents with TH
Number of participants, *n* (%)	15 (40.50%)	22 (59.50%)
Mean age (year), (SD)	14.86 (1.08)	15.33 (0.86)
Age range	13.04 ~ 16.72	13.76 ~ 17.44
Gender, male *n* (%)	8 (53.30%)	9 (40.90%)
Raven standard score	26.67 (5.84)	22.50 (4.84)
Socioeconomic status (SD)	−0.26 (0.66)	0.48 (0.41)
Communicate model, *n* (%)		
Oral	0 (0%)	
Sign	6 (40.00%)	
Both	9 (60.00%)	
Type of hearing aids, *n* (%)		
No hearing aids	4 (26.70%)	
Hearing aids	10 (66.70%)	
Cochlear implants	1 (6.60%)	
Hearing condition when born, *n* (%)		
No hearing loss	2 (13.30%)	
Hard of hearing	3(40.00%)	
Deaf	4 (26.70%)	
Unknown	3 (20.00%)	
Degree of hearing loss, *n* (%)		
Profound	7(13.30%)	
Severity	6(40.00%)	
Moderate	2(13.30%)	
Mild	0(0%)	

No significant differences were observed between the two groups regarding gender ratios, *χ*^2^ (1) = 0.552, *p* = 0.457, or mean ages, *t* (35) = 1.477, *p* = 0.149. However, paternal education levels (*Z* = 3.701, *p* < 0.001) and family annual income (*Z* = 2.359, *p* = 0.026) were significantly higher in the TH group compared to the HL group, while maternal education levels did not differ between the groups, *Z* = 1.129, *p* = 0.334. A socioeconomic status (SES) score was calculated by standardizing and averaging paternal and maternal education levels and family income.

### 2.2 Experimental design

The current study employed a 2 (between-person factor: hearing groups—HL, TH) × 2 (within-person factor: moral identity—moral, immoral) × 3 (within-person factor: emotions—happiness, neutral, anger) mixed factorial design.

### 2.3 Experimental materials

#### 2.3.1 Moral identity stimuli

Following the approach of Cui et al. (2016), we initially selected six moral identity words as priming stimuli. To enhance priming effects, we developed supplementary reading materials for each identity, based on established research on moral identity and its effects on cognition and behavior (Aquino and Reed, 2002; Hardy and Carlo, 2011). The initial set included three moral identities (“anti-epidemic hero,” “blood donor,” and “firefighter”) and three immoral identities (“terrorist,” “murderer,” and “human trafficker”), chosen to establish a clear moral contrast.

A pilot study was conducted to assess the moral valence and evocativeness of these identities. Based on the results, four well-matched identities (“blood donor,” “firefighter,” “murderer,” and “human trafficker”) were selected for the final experiment, while “anti-epidemic hero” and “terrorist” were excluded to ensure balance in moral salience and consistency in emotional engagement across conditions. The detailed evaluation procedure and statistical results can be found in the Supplementary materials (Section A).

Given the sensitivity of the selected negative identities, we carefully evaluated their appropriateness for adolescent participants. The study protocol was approved by the Ethical Review Board, and all materials were reviewed to ensure they were presented in a neutral and research-focused manner. Prior to participation, adolescents and their guardians were fully informed about the content, and a trained psychologist was available during the study to provide support if needed. Additionally, participants had the right to withdraw at any time if they felt uncomfortable.

#### 2.3.2 Facial emotion expression pictures

Seventy-five facial expression images were randomly selected from the Chinese Facial Expression Picture System (Gong et al., 2011). Anger and happiness were chosen for their clear valence contrast, high arousal, and moral relevance, as anger is associated with social conflict and moral transgressions, while happiness is linked to social bonding and moral reinforcement (Gilet and Jallais, 2011). The detailed evaluation process, including the pilot study procedure, statistical analyses, and final image selection criteria, can be found in the Supplementary materials (Section B).

### 2.4 Experimental procedure

**Figure 1:**
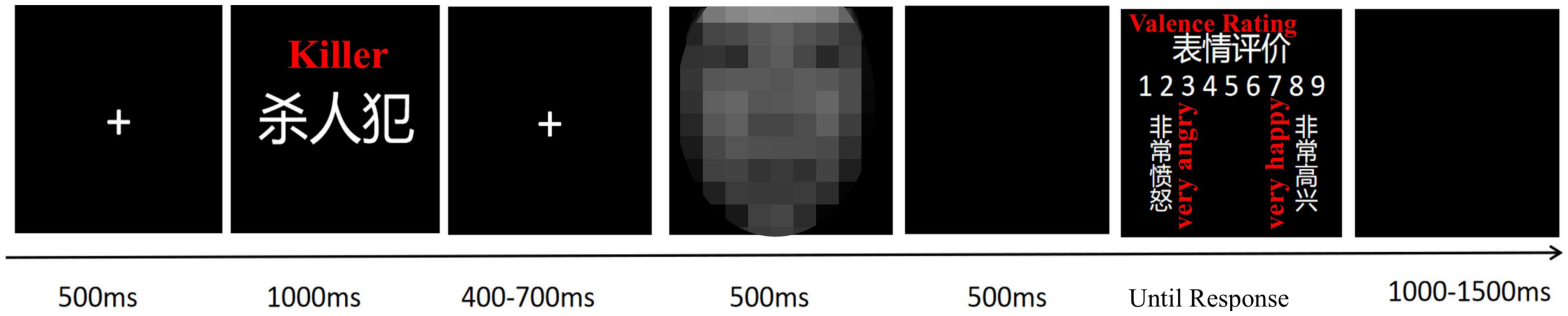
Procedures for the moral identity priming task.

The experiments were programmed using Experiment Builder 2.3.1. Each trial began with a fixation point (“+”) displayed for 500 ms, followed by the presentation of a moral identity prime (e.g., a moral word) for 1,000 ms. After a second fixation point (400–700 ms), a facial expression was displayed for 500 ms, followed by a blank screen lasting 500 ms. Participants then rated the emotion on a 9-point scale (1 = very angry, 9 = very happy). Each trial concluded with a blank screen lasting 1,000–1,500 ms (see Figure 1).

Participants first completed a practice session designed to familiarize them with the task. The practice phase required participants to repeatedly perform the task until they achieved at least 80% accuracy, ensuring they understood the instructions and response requirements. The practice trials began accumulating after participants had completed a minimum of 10 trials, and once they reached the required accuracy threshold, they proceeded to the formal experiment.

The experimental session consisted of 360 trials. In each trial, participants were instructed to associate the moral identity word with the presented face and rate the emotional expression. To ensure attention to the priming stimuli, 12.5% of the trials included an additional task requiring participants to identify the moral identity of the individual depicted in the image.

### 2.5 EEG recording

Electroencephalography (EEG) data were recorded using a 64-channel waveguard EEG cap (International 10–20 System) with Ag/AgCl electrodes, connected to an ANT Neuro device. Reference electrodes were positioned between Fpz and Fz, while ground electrodes were placed between Pz and Oz. Vertical electrooculogram (VEOG) data were recorded from a site above the left eye. Impedances were kept below 5 kΩ, and data were sampled at a rate of 1,000 Hz.

EEG data were preprocessed offline using the EEGLAB toolbox in Matlab (Delorme and Makeig, 2004). The data were re-referenced to the mastoid averages (M1, M2), resampled at 500 Hz, and band-pass filtered between 0.1 Hz and 30 Hz. Independent Component Analysis (ICA) was used to correct for eye-blink artifacts. ERP epochs time-locked to facial expression stimuli were extracted from 200 ms before to 1,000 ms after stimulus onset, with a 200 ms pre-stimulus baseline correction. Trials with amplitudes exceeding ±100 μV or contaminated by amplifier clipping were excluded. Participants with fewer than 30 valid trials in any condition were excluded from further analysis.

### 2.6 Time domain analysis

This study analyzed three ERP components: N170, N2, and LPP. Based on the topographic distribution of electrodes and prior empirical research (Dai et al., 2014; Shim et al., 2016), specific electrode sites were selected to index each component. The N170 was measured as the peak amplitude between 120 and 220 ms post-stimulus at electrodes P7, P8, PO7, and PO8 (Luo et al., 2010). The N2 was measured as the peak amplitude between 200 and 340 ms post-stimulus at electrodes Fz, F3, F4, FCz, FC3, and FC4 (Gu et al., 2019). The LPP was quantified as the mean amplitude between 300 and 500 ms post-stimulus at electrodes P1, P2, Pz, CP1, and CP2 (Syrjänen et al., 2018).

For statistical analyses, electrodes were grouped into three brain regions based on their scalp distribution. For N170, P7 and PO7 corresponded to the left hemisphere, while P8 and PO8 corresponded to the right hemisphere. For N2, the left region included F3 and FC3, the central region included Fz and FCz, and the right region included F4 and FC4. For LPP, the left region consisted of P1 and CP1, the central region was represented by Pz, and the right region included CP1 and CP2.

### 2.7 Statistical analysis

All statistical analyses were conducted using SPSS 27.0 (IBM Corp, Armonk, NY, United States) with a significance level of *p* < 0.05. Effect sizes were reported using partial eta squared (η^2^ₚ). A 2 (hearing groups: HL, TH) × 2 (moral identities: moral, immoral) × 3 (emotion categories: anger, neutral, happy) repeated-measures ANOVA was applied to behavioral (emotional valence ratings) and ERP (N2, N170, LPP amplitudes) data. For ERP analyses, brain region (left, central, right for N2/LPP; left, right for N170) was included as a within-subject factor. *Post hoc* comparisons were Bonferroni-corrected, and simple effect analyses followed significant interactions. Mauchly’s test assessed sphericity, with Greenhouse–Geisser corrections applied when violated. Means (M), standard deviations (SD), *F*-values, *p*-values, and η^2^ₚ were reported for all effects, including non-significant results for transparency.

### 2.8 Transparency and openness

This study adheres to the Transparency and Openness Promotion (TOP) Guidelines by offering comprehensive methodological details and ensuring data accessibility for replication. Specifically:

#### 2.8.1 Data availability

The anonymized datasets generated and analyzed during the current study are available from the corresponding author on reasonable request.

#### 2.8.2 Materials availability

All materials used for the study, including questionnaires, ERP equipment specifications, and stimuli (e.g., facial emotion expressions and moral identity primes), are available upon request.

#### 2.8.3 Preregistration

This study was not preregistered. However, the research protocol, including the study’s hypotheses and analysis plan, was reviewed and approved by the South China Normal University’s Ethical Review Board (protocol number: SCNU-PSY-2021-268).

#### 2.8.4 Analytic methods transparency

All statistical analyses, including the application of ERP methods and significance thresholds, are fully described in the Methods section. The analysis scripts used in the current study can be provided upon request.

ERP analysis distinguishes early-stage moral processing in TH adolescents versus later-stage effects in those with HL, indicating different neurocognitive pathways.

## 3 Results

### 3.1 Behavioral results

A repeated measures ANOVA was conducted to analyze the emotional valence scores. Significant main effects were observed for moral identities, *F* (1, 35) = 13.187, *p* = 0.001, η^2^ₚ = 0.274, and emotion categories, *F* (1.322, 46.255) = 224.272, *p* < 0.001, η^2^ₚ = 0.865. However, the main effect of hearing groups was not significant, *F* (1, 35) = 0.247, *p* = 0.622, indicating that adolescents with HL and those with TH did not differ in their overall evaluations of the valence of the three facial emotion categories.

The two-way interaction effect of hearing groups × emotion categories was significant, *F* (1.322, 46.255) = 4.308, *p* = 0.033, η^2^ₚ = 0.110. Simple effect analysis revealed no significant differences between the two groups in the valence ratings for angry (M_TH_ = 2.650, SD_TH_ = 0.174 vs. M_HL_ = 3.105, SD_HL_ = 0.215, *p* = 0.108) and neutral facial emotion expressions (M_TH_ = 4.822, SD_TH_ = 0.101 vs. M_HL_ = 4.884, SD_HL_ = 0.125, *p* = 0.700). However, adolescents with HL (M_HL_ = 6.810, SD_HL_ = 0.245) rated happy facial expressions as having lower valence than adolescents with TH (M_TH_ = 7.503, SD_TH_ = 0.198, *p* = 0.034).

**Figure 2:**
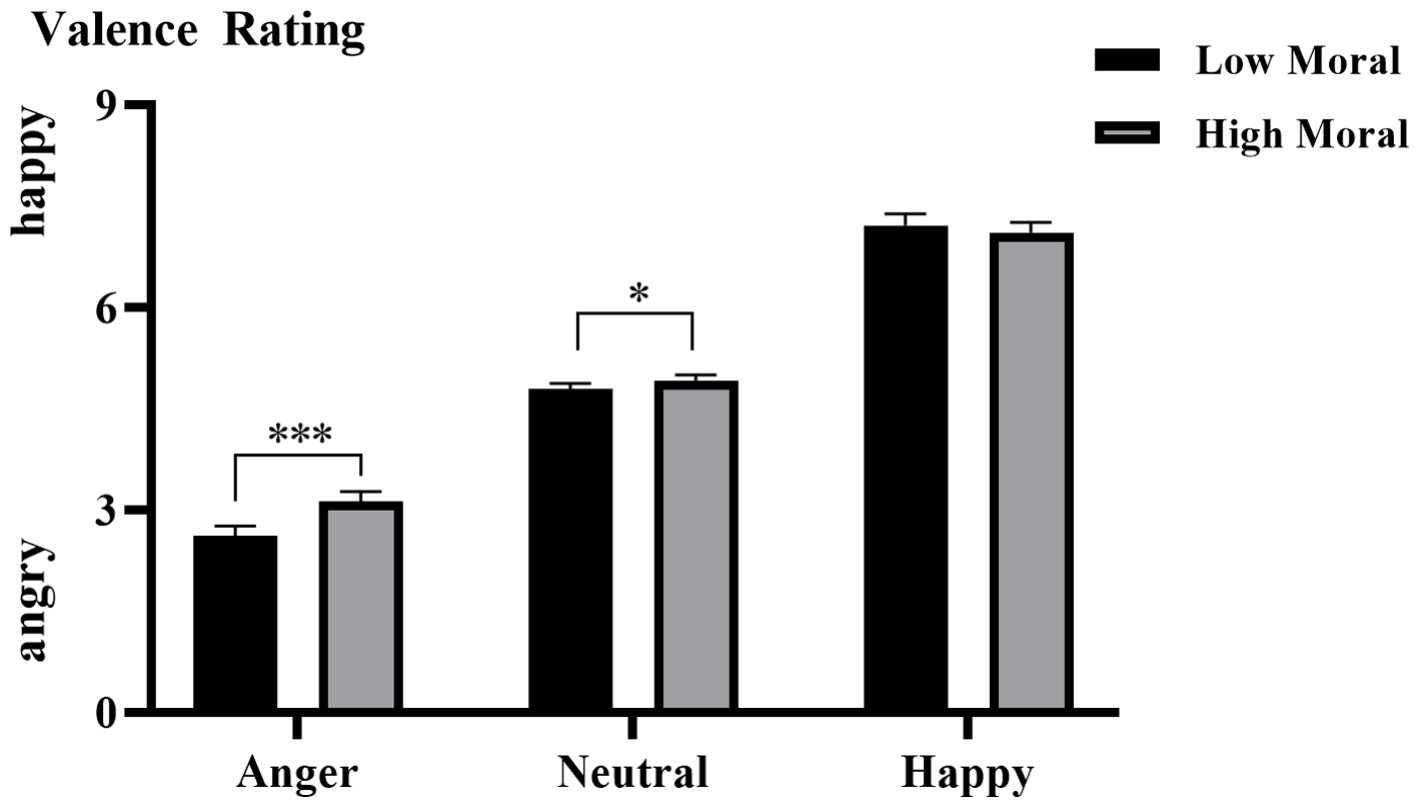
The averaged emotional valence of all the participants under each condition. Error bars represent SD, ^*^p < 0.05; ^***^p < 0.001.

The interaction effect of moral identities × emotion categories was also significant, *F* (1, 35) = 8.571, *p* = 0.006, η^2^ₚ = 0.197. As shown in Figure 2, simple effect analysis indicated that, compared to the immoral priming condition (*M* = 2.612, SD = 0.149), the moral priming condition (*M* = 3.131, SD = 0.143) resulted in less negative evaluations of angry faces (i.e., perceived as less angry; *p* < 0.001). A similar effect was observed for neutral faces, with higher valence ratings under the moral priming condition (*M* = 4.917, SD = 0.086) than under the immoral priming condition (*M* = 4.793, SD = 0.085, *p* = 0.018). However, no significant effect was found for happy faces (moral priming: *M* = 7.110, SD = 0.156 vs. immoral priming: *M* = 7.216, SD = 0.172, *p* = 0.103). The three-way interaction of hearing groups × moral identities × emotion categories was nonsignificant, *F* (1.787, 62.558) = 2.826, *p* = 0.073.

**Figure 3:**
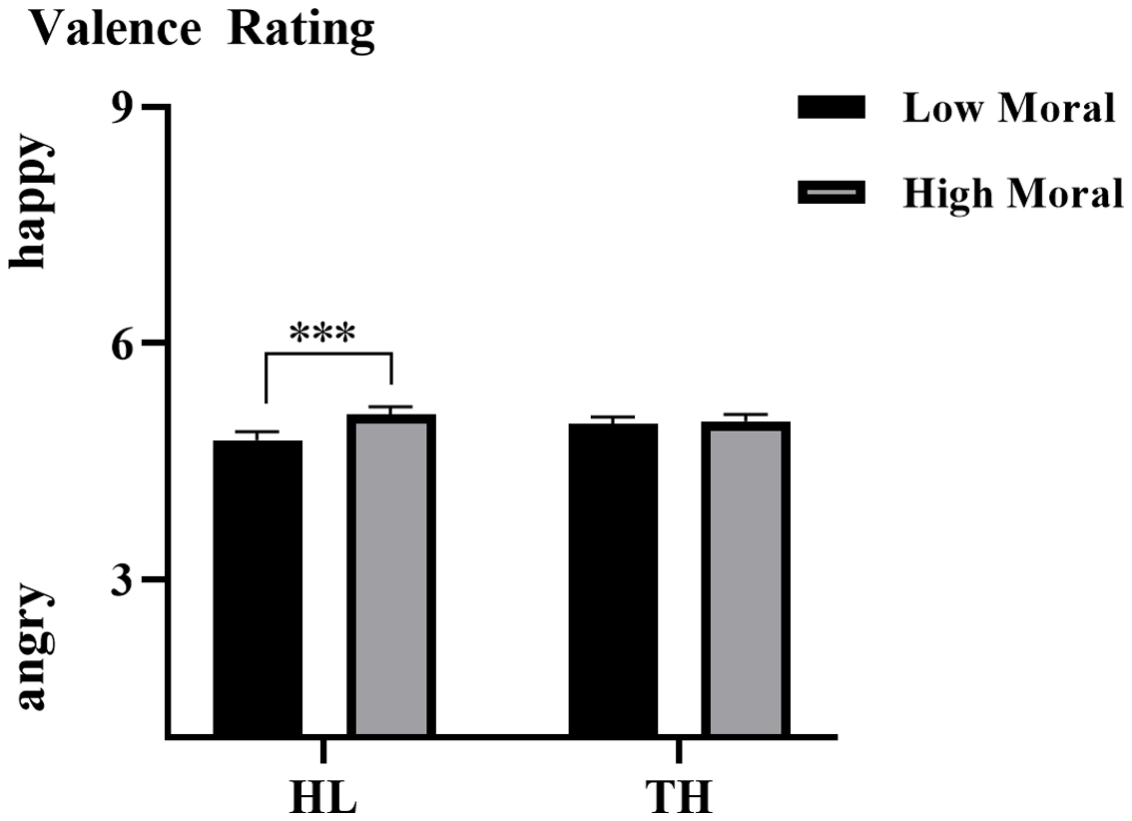
The averaged emotional valence of each group under each moral level condition. Error bars represent SD, ^*^p < 0.05; ^***^p < 0.001.

The interaction effect of hearing groups × moral identities was also significant, *F* (1, 35) = 8.571, *p* = 0.006, η^2^ₚ = 0.197. As illustrated in Figure 3, simple effect analysis showed a significant difference in emotional valence between the moral priming condition (*M* = 5.094, SD = 0.101) and the immoral priming condition (*M* = 4.771, SD = 0.104) for the HL group (*p* < 0.001). In contrast, no such difference was found for the TH group, where valence ratings were similar between the moral (*M* = 5.011, SD = 0.083) and immoral priming conditions (*M* = 4.977, SD = 0.086, *p* = 0.700).

### 3.2 ERP results

#### 3.2.1 N2

**Figure 4:**
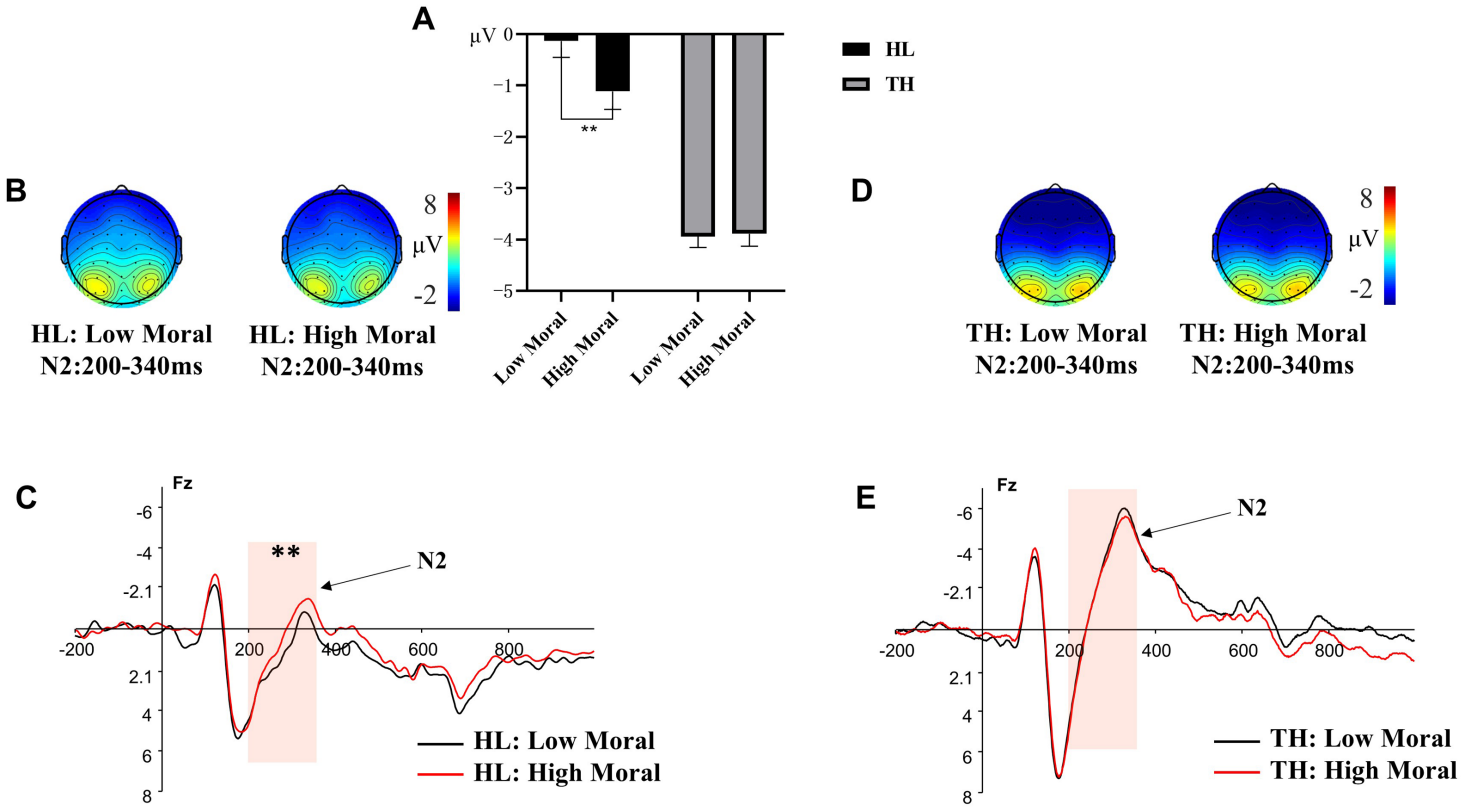
Different amplitude of N2 to pictures primed by different moral identity. **(A)** The averaged amplitude of N2 under each moral priming condition in the group of adolescents with hearing loss (HL) and the group of adolescents with typical hearing (TH). Error bars represent SD, ^*^p < 0.05; ^***^p < 0.001. **(B)** Topographic maps for the N2 effects in the 200-340 ms time window under each moral priming condition in the group of HL. **(C)** ERP responses time-locked to the onset of different expression pictures at Fz under different moral priming condition (Black: HL under high moral condition; Red: HL under low moral condition). The shaded 200-340 ms time window was used for the calculation of the peak amplitudes of the N2 in the group of HL. **(D)** Topographic maps for the N2 effects in the 200-340 ms time window under each moral priming condition in the group of TH. **(E)** ERP responses time-locked to the onset of different expression pictures at Fz under different moral priming condition (Black: TH under high moral condition; Red: TH under low moral condition). The shaded 200-340 ms time window was used for the calculation of the peak amplitudes of the N2 in the group of TH.

A 2 (hearing groups: HL, TH) × 2 (moral identities: moral, immoral) × 3 (emotion categories: anger, neutral, happy) × 3 (brain regions: left, central, right) repeated measures ANOVA was conducted on the amplitude of N2. The results indicated that the interaction effect of hearing groups × moral identities significantly predicted the N2 amplitude, *F* (1, 35) = 5.809, *p* = 0.021, ηₚ^2^ = 0.142. Further analysis of this interaction (see Figure 4) indicated that the difference in N2 amplitude between the moral priming condition (*M* = −1.115, SD = 1.354) and the immoral priming condition (*M* = −0.136, SD = 1.221) was significant for adolescents with HL (*p* = 0.005). However, this difference was not significant for adolescents with TH (moral priming: *M* = −3.887, SD = 1.118; immoral priming: *M* = −3.939, SD = 1.008, *p* = 0.848). Other two-way interaction effects, *F* < 2.886, *p* > 0.05, the three-way interaction effects, *F* < 2.207, p > 0.05, and the four-way interaction effect, *F* (3.258, 114.033) = 0.931, *p* = 0.434, were all nonsignificant.

In addition, the main effects of moral identities, *F* (1, 35) = 4.686, *p* = 0.037, η^2^ = 0.118, and emotion categories, *F* (2, 34) = 3.454, *p* = 0.043, η^2^ = 0.169, were significant. *Post hoc* tests revealed that the moral priming condition (*M* = −2.501, SD = 0.878) resulted in a more negative N2 amplitude compared to the immoral priming condition (*M* = −2.037, SD = 0.792, *p* = 0.037). Additionally, angry faces (*M* = −2.760, SD = 0.914) evoked a more negative N2 amplitude than happy faces (*M* = −1.940, SD = 0.812, *p* = 0.046). However, no significant differences were found between the N2 amplitudes evoked by neutral faces and happy faces (*p* > 0.05), or between neutral faces and angry faces (*p* > 0.05).

#### 3.2.2 N170

A 2 (hearing groups: HL, TH) × 2 (moral identities: moral, immoral) × 3 (emotion categories: anger, neutral, happy) × 2 (brain regions: left, right) repeated measures ANOVA was conducted on the amplitude of N170. The results revealed a significant main effect of brain regions, *F* (1, 35) = 14.177, *p* = 0.001, ηₚ^2^ = 0.288. *Post hoc* analysis indicated that facial emotion expression pictures evoked a more negative N170 amplitude in the right hemisphere (*M* = −3.190, SD = 0.710) compared to the left hemisphere (*M* = −0.687, SD = 0.627, *p* < 0.001). No other main effects or interaction effects were significant, *F* < 3.201, *p* > 0.05.

#### 3.2.3 LPP

A 2 (hearing groups: HL, TH) × 2 (moral identities: moral, immoral) × 3 (emotion categories: anger, neutral, happy) × 3 (brain regions: left, central, right) repeated measures ANOVA was conducted to analyze the amplitude of LPP. Significant main effects were observed for moral identities, *F* (1, 35) = 9.975, *p* = 0.003, ηₚ^2^ = 0.220, and brain regions, *F* (2, 70) = 15.344, *p* < 0.001, ηₚ^2^ = 0.305. However, the main effects of emotion categories, *F* (1.632, 57.128) = 0.383, *p* = 0.64, and hearing groups, *F* (1, 35) = 0.174, *p* = 0.679, were not significant.

Post hoc analyses indicated that for moral identities, the immoral priming condition (*M* = 6.344, SD = 0.850) elicited a more positive LPP amplitude compared to the moral priming condition (*M* = 5.746, SD = 0.857, *p* < 0.01). For brain regions, the central region (*M* = 6.804, SD = 0.891) showed a more positive LPP amplitude than both the right region (*M* = 5.872, SD = 0.871, *p* = 0.002) and the left region (*M* = 5.509, SD = 0.816, *p* < 0.001). The difference between the LPP amplitudes in the right and left regions was not significant (*p* = 0.685).

**Figure 5:**
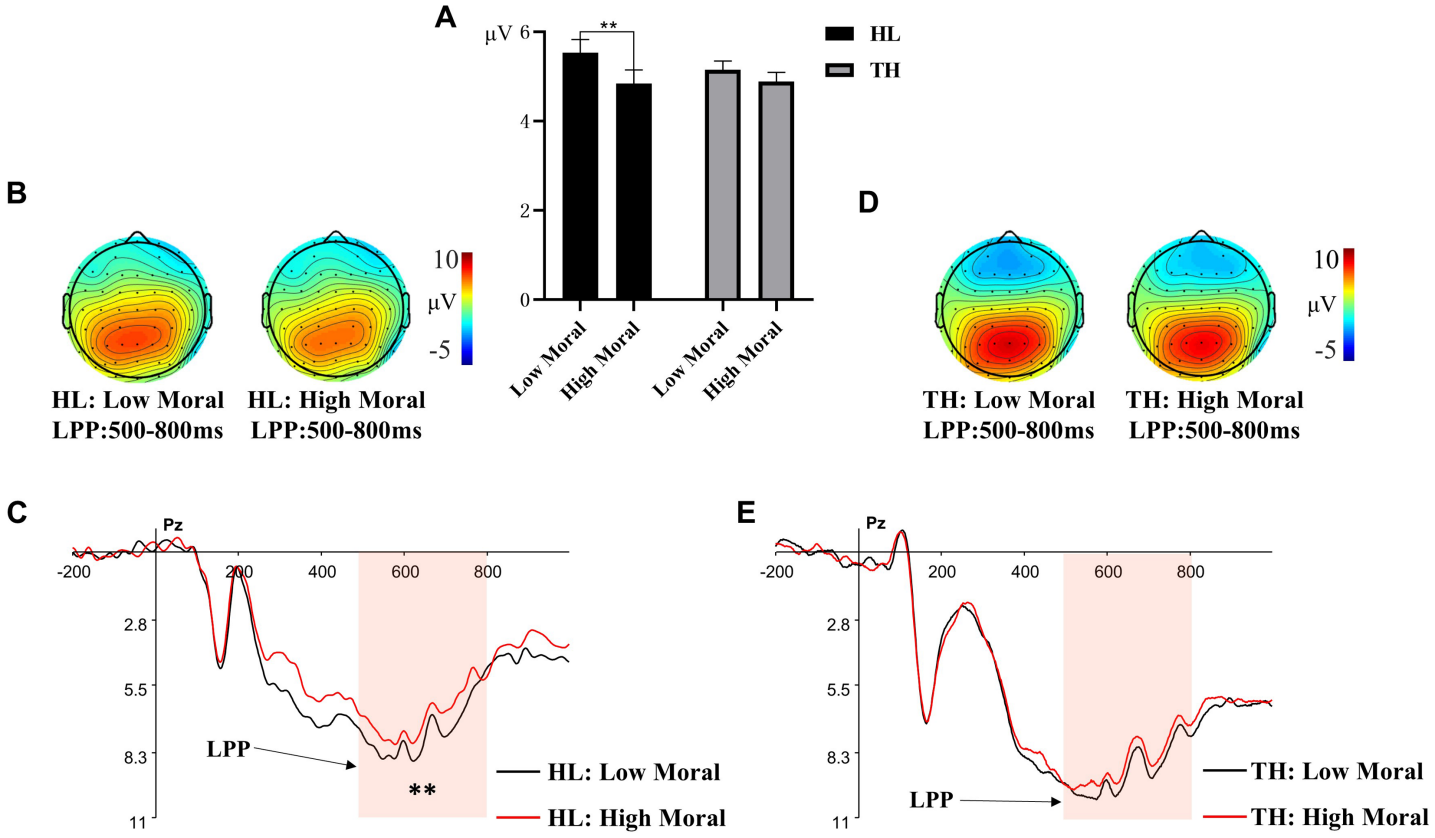
Different amplitude of LPP to pictures primed by different moral identity. **(A)** The averaged amplitude of LPP under each moral priming condition in the group of adolescents with hearing loss (HL) and the group of adolescents with typical hearing (TH). Error bars represent SD, ^*^p < 0.05; ^***^p < 0.001. **(B)** Topographic maps for the LPP effects in the 300-500 ms time window under each moral priming condition in the group of HL. **(C)** ERP responses time-locked to the onset of different expression pictures at Pz under different moral priming condition (Black: HL under high moral condition; Red: HL under low moral condition). The shaded 300-500 ms time window was used for the calculation of the mean amplitudes of the LPP in the group of HL. **(D)** Topographic maps for the LPP effects in the 300-500 ms time window under each moral priming condition in the group of TH. **(E)** ERP responses time-locked to the onset of different expression pictures at Pz under different moral priming condition (Black: TH under high moral condition; Red: TH under low moral condition). The shaded 300-500 ms time window was used for the calculation of the mean amplitudes of the LPP in the group of TH.

The interaction effect of hearing groups × moral identities was marginally significant, *F* (1, 35) = 3.479, *p* = 0.071, ηₚ^2^ = 0.220, suggesting a large effect size (see Figure 5). Simple effect analysis revealed that for adolescents with TH, there was no significant difference in LPP amplitude between the high moral priming condition (*M* = 6.279, SD = 1.091) and the low moral priming condition (*M* = 6.521, SD = 1.083, *p* > 0.05). However, for adolescents with HL, the low moral priming condition elicited a larger LPP amplitude (*M* = 6.167, SD = 1.312) than the high moral priming condition (*M* = 5.218, SD = 1.322, *p* = 0.003). No other main effects or interaction effects were significant, Fs < 1.431, ps > 0.05.

## 4 Discussion

This study employed a priming paradigm to explore how moral identity priming affects facial emotion processing in adolescents with HL. Findings from emotional valence ratings and ERP analyses indicated that moral identity priming significantly influenced facial emotion recognition in adolescents with HL, modulating neural responses in the N2 and LPP components. In contrast, adolescents with TH showed no substantial effects of moral identity priming. These results suggest that adolescents with HL process moral information differently during facial emotion recognition tasks, particularly in response to moral or immoral identity priming, compared to their TH peers.

These findings align with prior researches indicating that ERP components such as N2 and LPP reflect distinct stages of cognitive and emotional processing. The N2 component is particularly associated with conflict monitoring and selective attention (Calvo and Beltrán, 2013; Jost et al., 2022; Wang et al., 2025), consistent with our observation that adolescents with HL were more influenced by moral identity at this stage. This heightened effect likely results from reduced inhibitory control. In contrast, adolescents with TH, who generally demonstrate stronger inhibitory control, were better able to mitigate the effects of moral identity interference during the middle and later stages of emotional processing (Bouhours et al., 2021; Mancini et al., 2022).

The LPP component, linked to sustained attention and emotional evaluation, highlights the difficulties faced by adolescents with HL during facial emotion processing. The significant differences in LPP amplitudes between moral and immoral priming conditions in the HL group suggest that moral identity interfered with their ability to accurately evaluate facial expressions. This interference likely arises because adolescents with HL must allocate additional cognitive resources to resolve conflicting social cues (Balconi and Mazza, 2009; Hajcak et al., 2010). As a result, processing morally complex stimuli becomes more cognitively demanding for these adolescents, complicating their emotional evaluations.

### 4.1 Contrasting with current theories

Our findings provide a nuanced perspective on Bruce and Young’s (1986) parallel processing theory, which posits that facial emotion expressions and identity cues are processed independently. Adolescents with TH generally adhered to this model, exhibiting minimal interference from moral identity cues during facial emotion recognition. However, results for adolescents with HL align more closely with the interactive processing theory proposed by Ganel and Goshen-Gottstein (2004). This theory suggests that emotional and identity cues are processed interactively, particularly when cognitive control mechanisms, such as attentional allocation and inhibitory control, are weakened. Adolescents with HL demonstrated greater susceptibility to moral identity interference, likely due to deficits in these cognitive processes (Merchan et al., 2022; Shende et al., 2022). These findings indicate that when inhibitory control is compromised, as is often the case with HL, facial emotion processing becomes more vulnerable to identity-related cues, underscoring the interdependence of emotional and identity information in these conditions.

Posamentier and Abdi (2003) challenged the traditional notion of independent processing of facial expressions and identity, arguing that identity cues can modulate emotion recognition, particularly when these cues carry strong social or moral significance. Their research showed that facial emotion recognition is not entirely independent of identity-related information, especially under morally salient conditions. Our findings corroborate this view, especially in the HL group, where moral identity significantly influenced facial emotion processing during the middle (N2) and late (LPP) stages. In contrast, adolescents with TH were better at mitigating the influence of moral identity cues, suggesting that sensory or cognitive impairments, such as HL, amplify the interdependence of identity and emotion processing. This heightened sensitivity to moral identity interference in adolescents with HL likely stems from weakened inhibitory control mechanisms, which are critical for filtering out irrelevant social information during facial emotion recognition tasks.

### 4.2 The role of inhibitory control in adolescents with HL

The observed differences between the HL and TH groups can be attributed to the inhibitory control deficits frequently observed in adolescents with HL. Studies employing emotion-Stroop tasks have consistently shown that HL adolescents struggle to filter out irrelevant or conflicting emotional information (Chen et al., 2022). These inhibitory control challenges likely explain why HL adolescents are more susceptible to moral identity interference during facial emotion processing. This increased vulnerability demands greater cognitive and emotional resources, as evidenced by the heightened activation in the N2 and LPP stages.

These findings align with research by Merchan et al. (2022), which demonstrated that inhibitory control deficits impede HL adolescents’ ability to disregard complex social cues, intensifying their emotional processing demands. When confronted with conflicting or morally complex stimuli, these impairments amplify the cognitive load required for emotional regulation, further emphasizing the difficulties HL adolescents face in navigating social interactions.

### 4.3 Clinical implications

The findings from this study have important implications for the education and emotional management strategies of adolescents with HL. By identifying the challenges, they encounter in facial emotion recognition, educators and practitioners can design targeted interventions to enhance their ability to accurately interpret emotions, thereby reducing the likelihood of social conflicts.

Additionally, interventions aimed at strengthening cognitive control and inhibitory abilities hold promise for improving emotional regulation, particularly in complex social environments. Enhancing these cognitive functions may equip adolescents with HL to better navigate social interactions, alleviating the emotional burden associated with processing conflicting or nuanced social cues.

## 5 Conclusion

In summary, this study provides compelling evidence that moral identity exerts a more disruptive influence on facial emotion processing in adolescents with HL compared to their typical hearing peers. This disruption is reflected in both behavioral outcomes and ERP findings, particularly during the N2 and LPP stages. These results highlight the importance of investigating the interaction between moral and emotional information in individuals with sensory or cognitive deficits. Furthermore, the findings underscore the necessity of developing targeted interventions to enhance inhibitory control in adolescents with HL, potentially mitigating the adverse effects of moral identity interference on emotional processing.

## Constraints on generality

This study provides valuable insights into how adolescents with HL process facial emotion expressions in relation to moral identity; however, several limitations constrain the generalizability of the findings. First, the sample was drawn from a specific cultural context (China), which may not fully represent adolescents with HL from other cultural or socioeconomic backgrounds. Cross-cultural differences in moral values and socialization processes could influence how moral identity affects emotional processing. Second, the study focused exclusively on adolescents, meaning the findings may not generalize to other age groups, such as younger children or adults with HL. Third, participants varied in their levels of HL, and differences in communication methods (e.g., sign language, lip-reading) were not controlled, potentially affecting how emotional and moral cues were processed. Future research should examine how diverse communication modes and varying degrees of hearing impairment shape the interaction between moral identity and emotional processing across different populations.

Additionally, the study included negative moral roles (e.g., terrorists, criminals), which may involve socially sensitive content. Although all experimental materials were reviewed and approved by the ethics committee and underwent a suitability assessment prior to the experiment to ensure they would not cause psychological discomfort to participants, there remains a possibility that some adolescents may have experienced emotional reactions. However, no instances of discomfort or withdrawal due to the content of the materials were observed during the experiment. Future research could further refine the experimental materials or adopt moral roles more appropriate for adolescent participants to enhance the ecological validity and ethical appropriateness of the study context.

Finally, this study employed a specific set of facial emotion stimuli and moral identity primes, limiting the range of emotional expressions and moral contexts explored. Expanding future studies to include a broader array of stimuli and scenarios would provide a more comprehensive understanding of these processes. Specifically, future research could extend the investigation to additional basic emotions, such as sadness, surprise, fear, and disgust, to examine whether moral identity influences the recognition of these emotions in similar or distinct ways. This would offer deeper insights into the generalizability of our findings and the broader role of moral identity in emotional processing.

## Reflexive statement

This study investigated the influence of moral identity on facial emotion processing in adolescents with HL. Considering the broader implications, it is essential to acknowledge the potential social and ethical impact of these findings, particularly regarding policy and marginalized groups.

## Policy implications

The findings underscore the need for tailored interventions for adolescents with HL, particularly cognitive training programs designed to enhance their ability to process both moral and emotional cues. These insights have significant policy implications for inclusive education programs, emphasizing the importance of addressing the specific emotional processing needs of adolescents with hearing disabilities to create more equitable learning environments.

## Potential for misinterpretation or harm

There is a risk that these results could be misinterpreted, potentially reinforcing stigmatization of adolescents with HL by portraying them as emotionally or morally “deficient.” To avoid such harm, it is vital that these findings are not used to perpetuate negative stereotypes or exclude these individuals from social or educational opportunities. Future research and interventions must highlight the capabilities and strengths of adolescents with HL, focusing on their unique cognitive and emotional strengths rather than solely on challenges.

## Avoiding reinforcement of negative stereotypes

The study design intentionally avoids framing HL as a deficiency. Instead, it emphasizes HL as a factor influencing emotional processing in specific ways. Differences in processing moral and emotional cues are framed as opportunities to understand unique cognitive pathways and strengths that adolescents with HL may possess. This approach ensures that these differences are not misinterpreted as deficits.

## Researcher bias and worldview

The choice of this research topic reflects an awareness of gaps in existing literature regarding the emotional and moral processing of marginalized groups, such as individuals with HL. The study design was informed by values of inclusivity and social justice, aiming to deepen understanding of how social and cognitive processes interact in diverse populations. By addressing these gaps, the research seeks to contribute to reducing educational and social inequities.

By incorporating these reflexive considerations, this study aims to mitigate potential negative impacts or misinterpretations of its findings and promote equity-driven, inclusive research practices.

## Data Availability

The raw data supporting the conclusions of this article will be made available by the authors, without undue reservation.
