# Supplementary material for: The effect of moral identity on facial emotion processing in adolescents with hearing loss: an event-related potentials study
Source: Front Neurosci. 2025 Mar 26;19:1559627. doi: 10.3389/fnins.2025.1559627 (PMC11980441; doi:10.3389/fnins.2025.1559627)
Supplement: Supplementary file 1 [file Data_Sheet_1.docx]

**Section A**

**Moral identification stimuli**

**Good-Person Moral Identities**

**Anti-Epidemic Hero (“抗疫英雄”):** The COVID-19 outbreak in January 2020 served as a call to arms. Anti-epidemic heroes selflessly confronted the disease, leaving behind their children, parents, and loved ones. "For us, where we should be is where life is at risk," they declared, embodying courage and dedication in the face of unprecedented challenges.

**Blood Donor (“献血者”):** Fang, a 29-year-old patient at Jinan Maternal and Child Health Hospital, faced a critical emergency due to a sudden amniotic fluid embolism. She urgently required a large supply of AB blood. In response to the hospital's call for help through various media outlets, over 600 individuals rushed to donate blood. Their collective efforts ultimately saved Fang's life, bringing her out of danger and back to health.

**Firefighter (“救火英雄”):** When a fire broke out in a residential neighborhood in Zigong City at midnight, residents raced to escape. In this critical moment, a team of firefighters arrived swiftly and courageously moved toward the flames to extinguish the fire, safeguarding lives and protecting property.

**Bad-Person Immoral Identities**

**Terrorist (“恐怖分子”):** According to CCTV News, a group of terrorists in Kunming carried out a horrific massacre at a densely populated railway station as an act of revenge against society. Men, women, and children were tragically and brutally killed in this senseless attack.

**Murderer (“杀人犯”):** The “95” Shenzhen robbery and murder cases refer to a series of violent crimes committed by 16 individuals, including Zhang Xiaojian and Chen Weixiang, in the Bao’an District of Shenzhen between 1993 and 1995. The group targeted highway drivers traveling between Bao’an District and Shenzhen Airport, using seduction as a tactic to facilitate robbery and murder. The brutality of these crimes had a profoundly destructive impact on the harmony of society.

**Human Trafficker (“人口贩子”):** According to CCTV News, police apprehended over 30 individuals involved in a notorious human trafficking case. Tragically, when the rescued youngsters were located, it was revealed that they had suffered severe harm, resulting in permanent disabilities.

These supplementary materials were designed to reinforce the moral identity priming effect by providing context and real-world examples associated with each identity.

**Evaluation of Moral Stimuli**

**Selection of Moral Identity Words**

Following the approach of Cui et al. (2016), we initially selected six moral identity words as priming stimuli. To enhance the priming effects, supplementary reading materials were developed for each identity. Participants first read these materials and were then presented with the corresponding moral identity word. The initial identity set included:

**Moral identities**: “anti-epidemic hero,” “blood donor,” and “firefighter.”

**Immoral identities**: “terrorist,” “murderer,” and “human trafficker.”

**Pilot Study for Moral Identity Stimuli Validation**

To ensure the validity and suitability of these identity stimuli, a pilot study was conducted with 16 middle-school students (Mage = 14.875, SD = 0.957; including 9 males). Participants rated each identity on a 7-point scale based on two key dimensions:

**Moral valence** (1 = highly immoral, 7 = highly moral)

**Evocativeness** (1 = not evocative, 7 = highly evocative)

**Selection Criteria and Statistical Analyses**

Based on statistical analyses, we selected four identities that were well-matched in arousal (evocativeness) while differing in moral valence, ensuring comparability in the priming effect. The final set included:

Moral identities:

“Blood donor” (M = 6.688, SD = 0.176)

“Firefighter” (M = 6.688, SD = 0.198)

Immoral identities:

“Murderer” (M = 1.625, SD = 0.340)

“Human trafficker” (M = 2.125, SD = 0.473)

Statistical comparisons confirmed that: The moral valence ratings of the selected moral identities (blood donor vs. firefighter) did not significantly differ (p > 0.05). Similarly, the immoral identities (murderer vs. human trafficker) showed no significant difference in moral valence (p > 0.05). Evocativeness ratings (Murderer: 5.313, SD = 0.472; Human trafficker: 5.375, SD = 0.482; Blood donor: 5.438, SD = 0.398; Firefighter: 5.875, SD = 0.352) were also not significantly different across groups (p > 0.05).

These results confirm that the selected four moral identities were well-matched in arousal (evocativeness) while exhibiting distinct moral valence, making them suitable for moral identity priming.

**Exclusion of Certain Identity Stimuli**

Although “anti-epidemic hero” and “terrorist” were initially included, their moral valence and evocativeness ratings did not align well with the other moral and immoral stimuli. To ensure consistency and comparability, these two identities were excluded from the final experiment.

**Section B**

**Evaluation of emotion expression pictures**

To ensure the appropriateness of the facial stimuli used in the study, 75 facial expression images were randomly selected from the Chinese Facial Expression Picture System (Gong, Huang, Wang, & Luo, 2011). A pilot study was conducted with 19 middle school students (Mage = 15.37, SDage = 1.01; 10 males), who evaluated each image based on emotional valence, arousal level, and recognition accuracy. Emotional valence was rated on a 7-point scale (1 = very angry, 7 = very happy), and arousal level was also rated on a 7-point scale (1 = low arousal, 7 = high arousal). Participants also categorized each facial photograph by emotion type to assess recognition accuracy. Based on these ratings, 20 happy, 20 neutral, and 20 angry images were selected, ensuring gender balance across the images.

Among various emotional expressions, anger and happiness were chosen as the primary stimuli due to their clear valence contrast, high arousal levels, and moral relevance. Other emotions such as sadness, surprise, fear, and disgust were excluded due to their context dependency and weaker moral associations. Fear and disgust are primarily linked to survival-related threats rather than moral judgment, sadness is associated with loss rather than moral appraisal, and surprise is an ambiguous emotion that could introduce confounds (Taylor & Uchida, 2022). Anger, on the other hand, is associated with social conflict and moral transgressions, while happiness is linked to social bonding and moral reinforcement (Gilet & Jallais, 2011). Therefore, anger and happiness provided the most suitable framework for examining the effects of moral identity on facial emotion recognition.

To confirm the validity of the selected images, statistical analyses were conducted. A one-way ANOVA revealed a significant difference in emotional valence among the three emotion types, F(2, 57) = 163.231, p < .001, η² = 0.852. Happy faces (M = 6.961, SD = 0.652) were rated significantly higher in valence than neutral faces (M = 4.715, SD = 0.346) and angry faces (M = 3.213, SD = 0.883), with neutral faces also rated significantly higher than angry faces (p < .001). Arousal levels also differed significantly across the three emotions, F(2, 57) = 118.093, p < .0016, η² = 0.817, with happy and angry faces eliciting significantly higher arousal than neutral faces. However, recognition accuracy did not differ significantly among the three emotion types, F(2, 57) = 1.395, p = .257.

The statistical validation confirms that anger and happiness serve as strong, distinguishable emotional stimuli with clear valence and arousal differences, making them optimal for investigating the effects of moral identity on facial emotion recognition.
